# Breakfast and psychosocial behavioural problems in young population: The role of status, place, and habits

**DOI:** 10.3389/fnut.2022.871238

**Published:** 2022-08-23

**Authors:** José Francisco López-Gil, Lee Smith, Rubén López-Bueno, Pedro Juan Tárraga-López

**Affiliations:** ^1^Health and Social Research Center, Universidad de Castilla-La Mancha, Cuenca, Spain; ^2^Centre for Health, Performance and Wellbeing, Anglia Ruskin University, Cambridge, United Kingdom; ^3^Department of Physical Medicine and Nursing, University of Zaragoza, Zaragoza, Spain; ^4^Departamento de Ciencias Médicas, Facultad de Medicina, Universidad de Castilla-La Mancha, Albacete, Spain

**Keywords:** healthy diet, nutrition, lifestyle, mental health, preschoolers, children, adolescents

## Abstract

The aim of this study was to examine whether breakfast status, place and habits are associated with psychosocial behavioural problems in a nationally representative sample of young people aged 4–14 years residing in Spain. This study analysed secondary data from the Spanish National Health Survey (2017), including 3,772 Spanish children and adolescents. Breakfast status, place, and habits were assessed by *ad hoc* questions answered by parents/guardians. The Strengths and Difficulties Questionnaire (SDQ) parents’ version form was applied to evaluate the psychosocial health of their children. Skipping breakfast and eating breakfast out of home were linked to greater odds of psychosocial behavioural problems (skipping breakfast: OR = 3.29; CI 95%, 1.47–7.35; breakfast out of home: OR = 2.06; CI 95%, 1.27–3.33) than eating breakfast at home. Similarly, not consuming coffee, milk, tea, chocolate, cocoa, yogurt, etc., for breakfast was related to greater odds of psychosocial behavioural problems (OR = 1.76; CI 95%, 1.21–2.55). This association was also found for those who did not eat bread, toast, cereals, pastries, etc., for breakfast (OR = 1.31; CI 95%, 1.01–1.73). Conversely, not consuming eggs, cheese, ham, etc., was associated with lower odds of psychosocial behavioural problems (OR = 0.56; CI 95%, 0.38–0.83). Our results show that eating breakfast (specifically at home) and breakfast habits related to the intake of certain food/beverages groups were associated with higher or lower odds of psychosocial behavioural problems.

## Introduction

Psychosocial health is broadly defined to include psychological and social psychological outcomes interlinked with socioeconomic factors (1). There is no accepted definition in the field, although it usually includes characteristics such as self-esteem and mood, as well as affect, such as anxiety (2). Importantly, affective disorders (e.g., anxiety) are the leading causes of illness and disability (3), as well as years lost due to disability (4) among children and adolescents (young people). Most affective disorders begin in childhood (3) and have been shown to be considerably stable over time (5). Therefore, early identification and treatment of such complications is essential during this life period (3). Owing to this, the assessment of affective disorders in childhood has expanded over the last two decades (3, 6, 7).

One potentially important factor associated with an increased risk of psychosocial complications is lower adherence to a healthy diet. In a meta-analysis (8) including 14 studies and 399,550 participants, skipping breakfast was related to a higher risk of stress, depression, and psychological distress in all age groups, as well as anxiety in adolescents. Likewise, another systematic review found similar findings (9).

Consuming a healthy breakfast on a daily basis has been observed to have multiple beneficial effects on psychosocial and health behaviours, such as improved memory recall and cognitive function, as well as higher levels of physical activity, among others (10). Furthermore, O’Sullivan et al. (11) indicated that breakfast quality is a key factor in the interaction between lifestyle and psychosocial health during early adolescence. Concluding that in children, a high-quality breakfast should include cereals, low-fat milk or other dairy products, and fruit or fruit juice (12). However, the intake of whole fruit rather than fruit juices has been discussed because of the more conclusive evidence of the health benefits of whole fruit (13). Thus, the Spanish Society of Community Nutrition (14) indicates that an adequate breakfast should be composed of the triad: (1) dairy products (one glass of milk, one fresh yogurt or cheese); (2) cereals (bread, cookies, whole wheat bread, homemade pastries or breakfast cereals); and (3) fruit or natural juice. Furthermore, it could also be complemented on some occasions with other protein foods, such as eggs, ham, nuts, etc. Thus, it is not surprising that daily breakfast consumption along with adequate breakfast selections have been highlighted as an important public health message (15).

The association between eating breakfast and psychosocial health in young people has been previously studied (8, 11, 16, 17). However, to date, the association between breakfast place (i.e., at home, out-of-home) and breakfast habits in relation to psychosocial behavioural problems in young people remains unknown. Interestingly, a systematic review suggested that the social context (e.g., breakfast at home) plays a key role in breakfast consumption (18). Similarly, eating at home favours the accessibility and availability of different foods, in addition to the key sociocultural scenario that family meals represent, since they provide a setting in which parents/guardians often control children’s behaviours, interact with them, and imposes rules and expectations on them (19). Supporting this notion, a systematic review by Lachat et al. (20) has pointed out that eating out of home is a risk factor for greater energy and fat consumption and lower consumption of micronutrients. More specifically, in children, the consumption out of home (e.g., coffee shops/restaurants) seems to favour a greater consumption of energy-dense foods and a lower consumption of nutrient-rich foods (21). This consumption of meals out of home could translate into a lower quality of individual meals (e.g., breakfast) and/or global diet, which could influence (as mentioned above) the psychosocial health complications of young people (22). Therefore, it could be suggested that eating breakfast away from the home or skipping breakfast *per se*, as well as consuming a breakfast that does not follow the Spanish recommendations, may be related to psychosocial behavioural problems in young people. Considering these premises, the aim of this study was to examine whether breakfast status, place and habits are associated with psychosocial behavioural problems in a nationally representative sample of young people aged 4–14 years residing in Spain.

## Materials and methods

### Population sample and study design

A cross-sectional study was carried out using nationwide data from the Spanish National Health Survey (2017) (23). This survey was conducted by the Ministry of Health, Consumer Affairs and Social Welfare *and the* National Statistics Institute (24). The sampling framework involved non-institutionalised Spanish individuals (i.e., people who live in group quarters other than institutions, such as college dormitories, rooming houses, religious group homes, communes, and halfway houses). A three-stage sampling design was applied. The census section was the first stage, the households were the second-stage units, and the individuals were the third-stage units. Within each household, an adult (aged 15 or older) was chosen to complete the Adult Questionnaire, and if there were minors (from 0 to 14 years of age), a minor was randomly chosen to complete the Minors Questionnaire. Data on the minors were reported by the parents/guardians. The participants were informed about the survey methodology through an informative letter from the Ministry of Health, Consumer Affairs and Social Welfare describing the aims of the survey, the anonymous and voluntary nature of participation, and the visit of a qualified and authorised interviewer.

For the purpose of this study, the sample was restricted to individuals aged 0–14 years old (Minors Questionnaire). The sample originally consisted of 6,106 participants. As psychosocial behavioural problems were only evaluated in children and adolescents from 4 to 14 years, we excluded 1,502 participants under the age of 4 years. Moreover, 832 participants were removed due to missing data in relation to diet, weight or height or any covariate. Thus, the final sample included 3,772 (49.4% females) Spanish children and adolescents. Differences between the included and excluded samples can be found in Supplementary Table 1.

Anonymised data were obtained from the Ministry of Health, Consumer Affairs and Social Welfare (23). Following the Spanish regulations, no Ethics Committee approval was needed for this study due to the use of secondary data.

### Procedures

#### Breakfast status and breakfast place

Using the original question from the Spanish National Health Survey (2017), we determined breakfast status (eating breakfast/skipping breakfast) and breakfast place (eating breakfast at home/eating breakfast out of home): “Where does your child usually eat breakfast?.” The options varied from (a) “at home”; (b) “out of home”; and (c) “no breakfast.”

#### Breakfast habits

Breakfast habits were determined by the following question: What does your child usually eat for breakfast? This question was applied for five different food/beverage groups: (a) “coffee, milk, tea, chocolate, cocoa, yogurt, etc.”; (b) “bread, toast, cookies, pastries, etc.”; (c) “fruit and/or juice”; (d) “eggs, cheese, ham, etc.”; (e) “other foods.” These categories correspond to those originally designed for the Spanish National Health Surveys and were designed to report what has been defined in Spain as a complete breakfast: hot drink (e.g., milk, cacao), accompanied by a solid food (e.g., bread, toasts), and fruit/fruit juice (25).

#### Strengths and difficulties questionnaire

The Strengths and Difficulties Questionnaire (SDQ) (26) parents’ version form was applied for the evaluation of different behavioural, emotional, and social problems related to the psychosocial health of children and adolescents.^1^ In this study, the Spanish version of the SDQ, which has been previously validated in other studies (7, 27), was applied. The SDQ contains a total of 25 items with five different subscales: (a) “emotional problems”, (b) “conduct problems”, (c) “hyperactivity”, (d) “peer problems”; and (e) “prosocial behaviour.” A Likert-scale with three possible options (0: “not true”; 1: “somewhat true”; 2: “certainly true”) was applied. Furthermore, the score on each subscale varies from 0 to 10 points. The first four subscales (hyperactivity, emotional problems, conduct problems, and peer problems) were used to establish a total score of psychosocial behavioural problems. The original 3-band categorisation of SDQ included normal (0–13 points), borderline (14–17 points), or “abnormal” (17–40 points). For additional analyses, the SDQ score was dichotomised into (a) no psychosocial behavioural problems (normal and borderline) and (b) psychosocial behavioural problems (abnormal).

#### Covariates

Age, sex, region, and immigrant status were declared by the parents/guardians. Social class was categorised according to the reference person’s occupation. Height and weight were also reported by parents/guardians. These values were used to determine the body mass index (BMI), which was transformed into BMI z score (zBMI) following the sex- and age-criteria from the International Obesity Task Force (IOTF) (28). According to the zBMI, participants were categorised as “no excess weight” (“underweight,” and “normal weight”) and “excess weight” (“overweight,” and “obesity”). Physical activity was evaluated by a modified short version of the International Physical Activity Questionnaire (29), with only one specific question about engagement in physical activity during free time. The question has four possible options: (a) “no exercise” (free-time mainly engaged in sedentary behaviours such as watching television, reading, going to the cinema, etc.); (b) “occasional sport or physical activity”; (c) “physical activity several times a month”, and (d) “sports or physical training several times a week” (23). Recreational screen time was assessed by asking respondents for weekdays and weekends independently: How much time does your child typically spend on a weekday/weekend in front of a screen, including a computer, tablet, television, video, video game or cell phone screen?.” The possible options were (a) “nothing or almost nothing”; (b) “less than 1 h”; and (c) “1 h or more.” The proportion of the young population meeting the recreational screen time recommendation was determined through the World Health Organization international guidelines for children under 5 years old (30) and the Canadian guidelines on screen time for the young population (31). Sleep duration was assessed by the following question: “Approximately how many hours does your child usually sleep per day? (Including nap times).” The meeting of the sleep recommendation was determined by the National Sleep Foundation’s sleep duration guidelines (32). Global quality diet was assessed by the Spanish Health Eating Index (S-HEI) (33), which is an adapted version of the original Healthy Eating Index (HEI) (34). The S-HEI includes 10 food groups (vegetables, cereals, legumes, fruit, meat, dairy, sweets, soft drinks, cold meats, and variety of the diet) divided into five categories (“never or hardly ever,” “one time per week,” “from one to two times per week,” “more than three times per week, but not daily,” and “daily”) according to the frequency of food intake indicated within the guidelines of the Spanish Society of Community Nutrition (35). The score on each food group ranged from 0 to 10 points. The total score for the S-HEI was calculated by summing the frequency of intake from the different food groups (Supplementary Table 2). A higher S-HEI score denotes a greater adherence to the guidelines of the Spanish Society of Community Nutrition (i.e., global quality diet). The choice of these covariates was based on prior literature (2, 22, 36–39).

### Statistical analysis

Continuous information was depicted as the mean (standard deviation), while categorical information was displayed as a number (percentage). Normality assumption was tested by statistical procedures (Kolmogorov–Smirnov test), as well as graphical procedures (normal probability plot). The SDQ score did not meet the assumption of normality. Consequently, we opted for the bootstrapping technique as a reliable method to determine confidence intervals for measures of both central tendency and association, as well as robust assessments of standard errors. To assess the differences between the mean values of the SDQ score (dependent variable) across breakfast habits (independent variable), analyses of covariance (ANCOVAs) were applied. For the ANCOVAs performed, we used the following *a priori* parameters: effect size (*f*) = 0.10, alpha (α) error probability = 0.05, statistical power (1-β) = 0.95, number of dependent variables = 1, number of groups = 2, and number of covariates = 15. Thus, a sample size with 1,302 observations would be enough to reach high effect sizes. The effect sizes of different ANCOVAs performed were computed by omega squared (ω^2^). Preliminary analyses did not indicate significant interactions between sex or age group and breakfast status, place and habits and mean differences in SDQ (*p* > 0.05 for all). Thus, all analyses were performed including both sex (girls and boys) age groups (preschoolers, children, and adolescents) together to obtain increased statistical power. Binary logistic regression analyses were performed to determine the association of psychosocial behavioural problems (dependent variable) according to breakfast status, place, and habits (independent variables). Age, sex, region, social class, immigrant status, physical activity level, recreational screen time, sleep duration, and S-HEI score were included as potential covariates. Additionally, since for most participants, breakfast consisted of a combination of habits, the analyses were adjusted for the remaining different possible habits. For instance, the consumption of “coffee, milk, tea, chocolate, cocoa, yogurt, etc.” was further adjusted by the consumption of “bread, toast, cookies, pastries, etc.,” “fruit and/or juice,” “eggs, cheese, ham, etc.,” and “other foods.” Statistical analyses were conducted with SPSS 25.0 (IBM Corp, Armonk, New York, NY, United States). A *p*-value lower than 0.050 was applied to determine statistical significance.

## Results

Table 1 depicts the characteristics of the study participants. The final sample included 3,772 Spanish young people. According to parents/guardians, 98.9% of the analysed sample ate breakfast, of whom 95.8% ate breakfast at home. The consumption of bread, toast, cereals, pastries, etc., was the most reported habit for breakfast (94.5%) by parents/guardians. The SDQ mean score was 7.4 ± 5.1. Furthermore, 12.9% of the sample presented borderline/abnormal values for the SDQ score.

**TABLE 1:** Descriptive data of the study participants (*N* = 3,772).

Variables	M (SD)/*n* (%)	CI 95%
Age (years)	9.4 (3.2)	8.4–10.4
**Sex**		
Boys	1908 (50.6)	49.0–52.2
Girls	1864 (49.4)	47.8–51.0
**Immigrant status**		
Native	3591 (95.2)	94.5–95.9
Immigrant	181 (4.8)	4.1–5.5
**Social class**		
Class 1 (the highest)	507 (13.4)	12.4–14.5
Class 2	317 (8.4)	7.5–9.3
Class 3	756 (20.0)	18.8–21.3
Class 4	529 (14.0)	12.9–15.1
Class 5	1199 (31.8)	30.3–33.3
Class 6 (the lowest)	464 (12.3)	11.3–13.3
**Anthropometric data**		
Weight (kg)	37.5 (14.8)	37.0–38.0
Height (cm)	139.4 (20.6)	138.7–140.1
BMI (kg/m^2^)	18.6 (3.7)	18.5–18.7
BMI (z-score)	0.57 (1.37)	0.53–0.61
**Eating healthy**		
S-HEI^a^ (score)	70.0 (8.8)	69.7–70.3
**Movement behaviours**		
Physical activity (%, sports/physical training several times a week)	1276 (33.8)	32.3–35.3
Meeting screen time recommendation (%, yes)	1857 (49.2)	47.6–50.8
Meeting sleep duration recommendation (%, yes)	2921 (77.4)	76.1–78.8
**Breakfast status**		
Eating breakfast (%)	3731 (98.9)	98.6–99.2
Skipping breakfast (%)	41 (1.1)	0.8–1.4
**Breakfast place**		
At home (%)	3613 (96.8)	96.3–97.4
Out of home (%)	118 (3.2)	2.6–3.7
**Breakfast habit**		
Coffee, milk, tea, chocolate, cocoa, yogurt, etc. (%, yes)	3563 (94.5)	93.7–95.2
Bread, toast, cookies, cereals, pastries, etc. (%, yes)	3330 (88.3)	87.3–89.3
Fruit and/or juice (%, yes)	780 (20.7)	19.4–22.0
Eggs, cheese, ham, etc. (%, yes)	197 (5.2)	4.5–5.9
Other foods (%, yes)	53 (1.4)	1.0–1.8
**Psychosocial behavioural problems**		
SDQ (score)^b^	7.4 (5.1)	7.2–7.6
Normal (%)	3295 (87.0)	86.3–88.4
Borderline (%)	259 (6.8)	6.1–7.7
Abnormal (%)	232 (6.1)	5.4–6.9

BMI, body mass index; SDQ, strengths and difficulties questionnaire; S-HEI, Spanish healthy eating index.

^a^Spanish Healthy Eating Index ranges from 0 to 100 points (33).

^b^Strengths and Difficulties Questionnaire ranges from 0 to 40 points (26).

**FIGURE 1:**
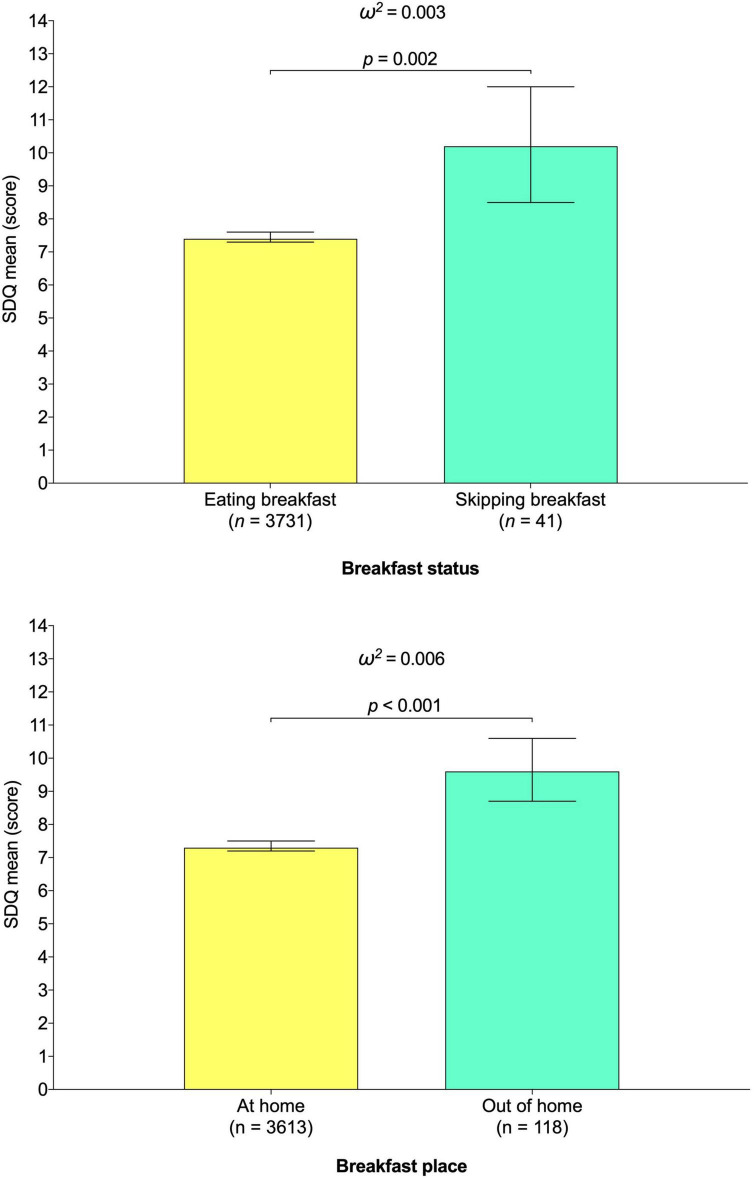
Association between breakfast status and breakfast place and Strengths and Difficulties Questionnaire mean score in young population. Estimated mean (bars) and 95% CIs (lines) represent values after adjustment for age, sex, region, social class, immigrant status, excess weight, physical activity level, recreational screen time, sleep duration, Spanish Healthy Eating Index score, and breakfast habits.

The mean differences in SDQ scores in relation to breakfast status and breakfast place are shown in Figure 1. A higher SDQ mean score was found for those who skipped breakfast in comparison with those eating breakfast (*p* < 0.001), with a small effect size (ω^2^ = 0.006). Similarly, this greater SDQ mean score was found in those who ate breakfast out of home compared to those who breakfast at home (*p* < 0.001), with a small effect (ω^2^ = 0.008).

**FIGURE 2:**
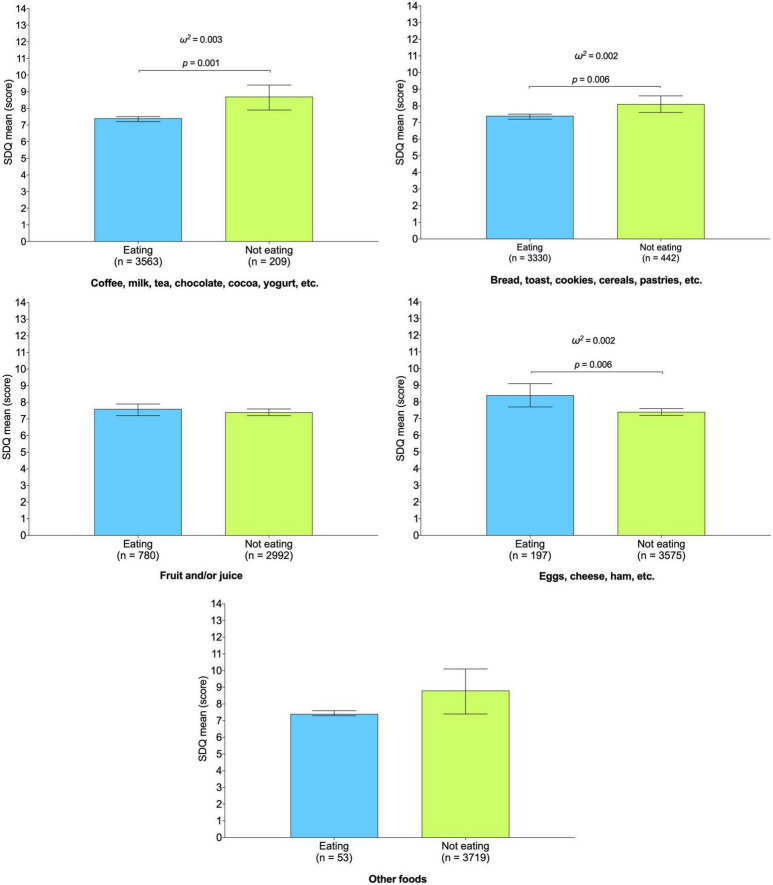
Association between different breakfast habits and Strengths and Difficulties Questionnaire mean score in young population. Estimated mean (bars) and 95% CIs (lines) represent values after adjustment for age, sex, region, social class, immigrant status, excess weight, physical activity level, recreational screen time, sleep duration, and Spanish Healthy Eating Index score. Analyses were also adjusted for the remaining possible habits (i.e., the consumption of “coffee, milk, tea, chocolate, cocoa, yoghurt, etc.” was further adjusted for “bread, toast, biscuits, pastries, etc.,” “fruit and/or juice,” “eggs, cheese, ham, etc.”, and “other foods.”

In relation to the mean differences between the SDQ score according to the different foods/beverages, Figure 2 shows that those who do not consume coffee, milk, tea, chocolate, cocoa, yogurt, etc., or bread, toast, cereals, pastries, etc., for breakfast showed a greater SDQ mean score (*p* < 0.05 for both). The effect sizes for both associations were small (coffee, milk, tea, chocolate, cocoa, yogurt, etc.: ω^2^ = 0.006; bread, toast, cereals, pastries, etc.: ω^2^ = 0.003). In addition, those consuming eggs, cheese, ham, etc., for breakfast showed higher SDQ mean scores (*p* = 0.006), with a small effect (ω^2^ = 0.003).

**FIGURE 3:**
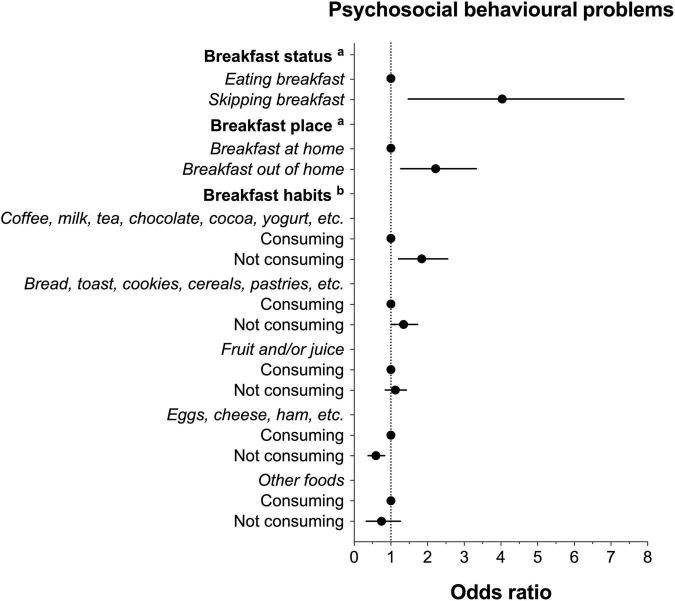
Odds of psychosocial behavioural problems according to breakfast status, place and habits. Data expressed as odds ratio (dots) and standard error (bars). No psychosocial behavioural problems group was considered as the reference category. Adjusted by age, sex, region, social class, immigrant status, excess weight, physical activity level, recreational screen time, sleep duration, and Spanish Healthy Eating Index score. ^a^Analyses were further adjusted by breakfast habits. ^b^Analyses were also adjusted for the remaining possible breakfast habits (i.e., the consumption of “coffee, milk, tea, chocolate, cocoa, yoghurt, etc.” was further adjusted for “bread, toast, biscuits, pastries, etc.,” “fruit and/or juice,” “eggs, cheese, ham, etc.,” and “other foods”).

On the other hand, Figure 3 shows the association between breakfast status, place or habits and psychosocial behavioural problems. Skipping breakfast and eating breakfast out of home were linked to greater odds of psychosocial behavioural problems (skipping breakfast: OR = 3.29; CI 95%, 1.47–7.35; breakfast out of home: OR = 2.06; CI 95%, 1.27–3.33) than eating breakfast at home. Similarly, not consuming coffee, milk, tea, chocolate, cocoa, yogurt, etc., for breakfast was related to greater odds of psychosocial behavioural problems (OR = 1.76; CI 95%, 1.21–2.55), with a small effect size ω^2^ = 0.006). This association was also found for those who did not consume bread, toast, cereals, pastries, etc., for breakfast (OR = 1.31; CI 95%, 1.01–1.73). Conversely, not consuming eggs, cheese, ham, etc., was associated with lower odds of psychosocial behavioural problems (OR = 0.56; CI 95%, 0.38–0.83), with a small effect size (ω^2^ = 0.003).

## Discussion

To the best of our knowledge, the present study is the first to assess the role of breakfast status, place and habits related to the intake of certain food/beverages groups and psychosocial behavioural problems in a young population. In the present study, we found that skipping breakfast, eating breakfast out of home, and eating some breakfast habits were associated with higher or lower odds of psychosocial behavioural problems in the young population after adjustment for several sociodemographic, anthropometric, and lifestyle factors.

One interesting finding is that skipping breakfast was associated with higher odds of psychosocial behavioural problems. Similarly, an association between an unhealthy diet and psychosocial behavioural problems has been suggested in the young population (8, 9), as well as in other age stages (8). Recently, a meta-analysis by Mullan and Singh (18) found that skipping breakfast was positively associated with odds of depression, stress and psychological distress in all age groups and anxiety in adolescence. Richards and Smith (40) showed that not eating breakfast daily was linked to psychosocial behavioural problems (e.g., anxiety, stress, depression). Despite this evidence, there are no well-defined mechanisms that explain why skipping breakfast might affect psychosocial health (8). Two possible explanations could partly justify this finding. First, a young population skipping breakfast might not be able to obtain the nutrients lost with the rest of the meals of the day (41). Second, children and adolescents who skip breakfast may compensate for their daily energy intake by consuming more energy-dense foods during the rest of the day or during lunch (42). These two factors could lead to an overall unhealthy/low-quality diet, which has been associated with depression or poorer psychosocial health in the young population (9, 22). Accordingly, breakfast consumption, as a part of healthy eating habits, may be promoted as a helpful approach to prevent psychosocial health problems (8).

Another interesting finding of this study is that young people who eat breakfast out of home showed higher odds of psychosocial behavioural problems than those who eat breakfast out of home. One reason explaining (at least partially) our results may be related to family meals. The social context (e.g., breakfast at home) may play a key role in breakfast intake, as it appears to be associated with higher quality breakfast intake (i.e., including dairy, cereal, and fruit groups) (18, 43). Children and adolescents who eat breakfast at home are more likely to do so in the presence of their family members. Thus, Kameyama et al. (44) showed that children (aged 7–12 years) who ate breakfast with their families less than once a week and those who ate breakfast alone on weekends showed a greater prevalence of borderline or abnormal psychosocial health status than those who ate breakfast seven times a week and those who ate breakfast on weekends with their families, respectively. Furthermore, one study by Videon and Manning (45) indicated that adolescents who regularly used to eat out of home/missed family meals show a greater prevalence of skipping breakfast, as well as a lower quality diet (e.g., low intake of dairy products). Similarly, Agathão et al. (46) pointed out the key protective role of regular family meals for psychosocial health. This is because family meals can facilitate parents to connect emotionally with children through feelings of closeness and belonging (47), as well as identify early changes in existing behavioural patterns (e.g., dress, friendships and academic performance) that may be associated with behavioural modification (48). In addition, family meals are a family time that provides an opportunity for families to connect despite the ongoing intense demands of modern life (49). Thus, current evidence indicates positive relations between diet quality and physical, emotional and mental strength in the young population, suggesting, as a promising strategy, the promotion of family-based meals, with a focus on breakfast. One possible reason justifying this relationship is that family meals (e.g., breakfast) could offer a formal/informal time in which parents/guardians could connect with their children’s emotional well-being (50), control children’s behaviours, or establish norms and restrictions (19). Another possible reason could lie in the relationship between breakfast out of home and a lower-quality breakfast/global diet. Eating out of home has been related to energy-dense and high-fat food consumption, as well as a lack of micronutrients (20). This association has also been shown in children (21). All of these aspects are associated with a lower quality breakfast/global diet, which has been associated with psychosocial behavioural problems among young people (22). Therefore, it seems reasonable to promote breakfast at home, preferably with the family and in a relaxed atmosphere, since family members have a great influence on the acquisition of habits among the young population (51).

On the other hand, we found that breakfast habits related to the intake of certain food/beverages groups were associated with lower (e.g., not consuming eggs, cheese, ham) or higher odds of psychosocial behavioural problems (e.g., not consuming coffee, milk, tea, chocolate, cocoa, yogurt, or not consuming bread, toast, cereals, pastries). With the questions provided by the Spanish National Health Survey, we are not able to know what and how many foods are consumed within a food group. For instance, it is possible that young people including in the group “coffee, milk, tea, chocolate, cocoa, yogurt, etc.” consume milk or dairy products and not necessarily coffee. In fact, coffee consumption is not recommended for Spanish young people (14), and its intake is low among this population (52). Supporting this idea, the latest breakfast recommendations in Spain separate coffee from the group “milk or dairy products” and include it in the group “other foods” (51). Given the impossibility of separating foods from the established groups, future editions of the national health survey should provide more specific questions regarding the breakfast composition of the young population. Nevertheless, breakfast habits can potentially impact psychosocial behavioural problems through several pathways. In this sense, there are dietary benefits of consuming breakfast, specifically if it includes cereals, grains, lower fat milk, and fruit/fruit juices, in comparison to the potential negative impact of skipping breakfast (53). This is mainly because they are suitable nutrient sources that may influence brain function, including carbohydrates, calcium, B-complex vitamins (including folate), dietary fibre, and iron (11). Supporting this notion, there is emerging evidence of the relationship between breakfast cereal intake and higher feelings of well-being (54) (although further research is necessary). Likewise, whole grains are rich in several macronutrients, including magnesium that may have beneficial effects on psychosocial health (55). In addition, Ferrer-Cascales et al. (56) revealed that a high-quality breakfast, characterised by the intake of cereal and dairy products, is related to a higher health-related quality of life and lower levels of perceived stress and depressive symptoms in adolescents. Similarly, adolescents eating a high-quality breakfast (e.g., cereals, milk) had an improved overall dietary pattern compared with their counterparts eating a low-quality breakfast (57). Similarly, children who ate breakfast had greater daily protein and energy consumption than children who skipped breakfast (58). Concerning psychosocial behavioural problems, it has been shown that the type of breakfast or lunch was associated with significant differences in well-being scores (59) and high levels of quality of life compared with those who eat a low-quality breakfast (in children) (60). Furthermore, O’Sullivan et al. (11) found in adolescents that for every additional food group eaten at breakfast, the associated total mental health score decreased after adjustment for potential confounding factors. The beneficial influence of an adequate quality breakfast (as a healthy lifestyle indicator) is especially important during childhood and adolescence, when dietary and other lifestyle habits begin to be acquired, resulting in long-term health and nutritional advantages in adulthood (60). Thus, the prevention of psychosocial behavioural problems in young populations supports the prevention of the onset in adulthood, suggesting that promoting a healthy breakfast (at home if possible) as a modifiable factor could be effective in preventing such problems (8).

Although the mechanisms through which breakfast habits contribute to decreased psychosocial behavioural problems remain unclear, certain mechanisms have been suggested. Thus, after eating breakfast, carbohydrates from foods (e.g., cereals, milk) are transformed into glucose, generating alterations in the levels of insulin, glutamate, acetylcholine, serotonin, and cortisol (11, 61). Carbohydrate intake is especially helpful for the brain after night fasting since it diminishes the production of cortisol levels, thereby reducing the “stress” signal (62). Furthermore, the transformation of carbohydrates into glucose is crucial for tryptophan formation, a precursor protein involved in the synthesis of serotonin, which regulates depressive symptoms, cognitive functioning, and irritable mood (63). Similarly, the consumption of tryptophan-rich foods has been noted as important to maintain a high quality of sleep and morning-type diurnal rhythm and indirectly improved psychosocial health, probably by the metabolism of tryptophan to serotonin in the daytime and melatonin at night in children (64). In contrast, the potentially beneficial effect of other vitamins (e.g., vitamin D) on psychosocial health in children has been pointed out in a recent systematic review (65). Additionally, it has been suggested that lower vitamin D levels may be linked to depression among children and adolescents (66). Thus, the consumption of milk and/or dairy products may provide greater amounts of vitamin D and, consequently, help to reduce the odds of psychosocial behavioural problems. In addition, B-complex vitamin deficiency (e.g., folate, B6, and B12) might also affect psychosocial behavioural problems, and it has been associated with mood and cognitive performance (67). Similarly, dietary patterns have been related to depression *via* alterations in folate and vitamin B12 serum levels (68). Moreover, Esnafoglu and Ozturan (66) has indicated that vitamin B12 and increased homocysteine may support the etiopathogenesis of depression. In addition, dietary fibre intake could also help in psychosocial behavioural problems. A meta-analysis of observational studies by Fatahi et al. (69) showed that a greater intake of total dietary fibre was linked to lower odds of depression, suggesting some possible mechanisms that could explain this association, such as the variations in the intestinal microbiome composition and the decrease in oxidative stress. Supporting this notion, one possible hypothesis is that a higher consumption in fibre-rich healthy foods (e.g., bread, cereals) may (to some degree) explain the association found. Another finding is the association found between the non-consumption of high-protein foods (e.g., eggs, cheese, ham) and lower odds of psychosocial behavioural problems. One possible explanation of this finding may be related to the ratio of carbohydrates/protein in the dietary intake. A higher intake of high-protein foods (e.g., eggs, cheese, ham) might displace carbohydrate-rich foods and modify the ratio of protein to carbohydrate, which has been related to consistent and reciprocal changes in important regulatory factors (e.g., cortisol). Despite this fact, it must be considered that the intake of some protein-rich foods (e.g., eggs) contains high amounts of choline that is essential for producing neurotransmitters that can positively affect psychosocial health (70). Based on the above, recommending what is considered a healthy breakfast in Spain [i.e., hot drink (e.g., milk, cacao), accompanied by a solid food (e.g., bread, toasts), and fruit/fruit juice (25)], could be useful for the prevention of psychosocial problems in young people.

This current study includes some limitations that should be noted. First, due to the cross-sectional design of this study, we cannot establish whether the observed relationships imply cause and effect associations. Longitudinal studies are necessary to determine how breakfast status, place and habits related to the intake of certain food/beverages groups could exert an essential role on psychosocial behavioural problems. Notwithstanding, this cross-sectional analysis could serve as a helpful first step in detecting relations between breakfast patterns and psychosocial behavioural problems in youth. Second, this study did not consider the influence of both daily energy intake and parental feeding practices, since there was no information available on both variables for analyses. Future studies using 24-h dietary recall or dietary history are needed to obtain more accurate information. However, it is complex to use more specific methodologies in relation to dietary intake or parental feeding practices in national epidemiological studies. Third, we were not able to establish individual associations between the consumption of a specific food and psychosocial behavioural problems, since frequencies and serving sizes of consumption of different breakfast items were not assessed. However, the Spanish National Health Survey (2017), as well as previous waves, were designed to report information related to a typical Spanish complete breakfast (25). Fourth, we used parent-reported questionnaires. For this reason, both measurement and recall bias are still plausible. Nonetheless, information was reported by parents/guardians and not by young people, which may be closer to the reality of their children’s breakfasts. Likewise, the SDQ and HEI are validated and useful instruments that have been widely used in the scientific literature for both psychosocial behavioural problems and diet quality. Fifth, BMI and excess weight were determined through height and weight reported by the parents/guardians for children and adolescents, which could introduce measurement error. Conversely, the main strength of this study is that, to date, it is the first study to examine the relationship between breakfast status, place and habits related to the intake of certain food/beverages groups and the association of psychosocial behavioural problems among a young population. Another strength is the nationwide, large sample of children and adolescents analysed. It is noteworthy that the relationship between breakfast (as an isolated meal) and the odds of psychosocial behavioural problems has been assessed. Therefore, it should be considered that a child or adolescent may not consume some of the foods listed for breakfast (e.g., fruits) and consume them later in the day at other meals. To try to minimise this concern, we adjusted the analyses performed by global quality diet (through the S-HEI score). Despite this fact, caution is required to interpret our results.

## Conclusion

Our results show that skipping breakfast or eating breakfast out of home is associated with higher odds of psychosocial behavioural problems in a nationwide, large sample of Spanish children and adolescents. Similarly, some habits related to the consumption of certain foods/beverages are related to higher or lower odds of psychosocial behavioural problems. This finding is clinically meaningful, as psychosocial behavioural problems are one of the most important worldwide worries in the young population. It might be possible to underscore the significance of focussing not only on breakfast intake but also on habits related to the consumption of certain foods/beverages to try to reduce the deleterious effects of psychosocial behavioural problems in young populations. Nevertheless, further studies with different designs are needed to verify cause-effect associations.

## Data availability statement

Publicly available datasets were analysed in this study. This data can be found here: https://www.sanidad.gob.es/estadisticas/microdatos.do.

## Ethics statement

Ethical approval was not provided for this study on human participants because following the Spanish regulations, no ethics committee approval was needed for this study, due to the use of secondary data. Written informed consent to participate in this study was provided by the participants’ legal guardian/next of kin.

## Author contributions

JL-G: conceptualisation, software, validation, formal analysis, and data curation. JL-G and LS: writing—original draft preparation. LS, RL-B, and PT-L: writing—review and editing. All authors have read and agreed to the published version of the manuscript.

## Footnotes

1
www.sdqinfo.org
