# Supplementary material for: Breakfast and psychosocial behavioural problems in young population: The role of status, place, and habits
Source: Front Nutr. 2022 Aug 23;9:871238. doi: 10.3389/fnut.2022.871238 (PMC9445130; doi:10.3389/fnut.2022.871238)
Supplement: Supplementary file 1 [file Table_1.pdf]

Table S1. Characteristics and differences between study participants that were included or not in the final analysis.

Variables	Total sample		Excluded sample		Analyzed sample		*p*
	N	n (%) / M (SD)	n	n (%) / M (SD)	n	n (%) / M (SD)	
Age	6106	7.6 (4.3)	2334	4.6 (4.2)	3772	9.4 (3.1)	<0.001
Infants (<1 y)	6106	256 (4.2)	2334	256 (11.0)	-	-	
Toddlers (1-2 y)	6106	768 (12.5)	2334	768 (32.9)	-	-	
Preschoolers (3-5 y)	6106	1095 (17.9)	2334	564 (24.2)	3772	531 (14.1)	<0.001
Children (6-12 y)	6106	3486 (57.1)	2334	658 (28.2)	3772	2828 (75.0)	
Adolescents (13-14 y)	6106	501 (8.2)	2334	88 (3.8)	3772	413 (10.9)	
Sex							
Boys	6106	3174 (52.0)	2334	1266 (54.2)	3772	1908 (50.6)	0.005
Girls	6106	2932 (48.0)	2334	1068 (45.8)	3772	1864 (49.4)	
Immigrant status							
Native	6106	5646 (92.5)	2334	2196 (94.1)	3772	1812 (95.0)	0.057
Immigrant	6106	460 (7.5)	2334	138 (5.9)	3772	96 (5.0)	
SES					3772		
Class 1 (the highest)	5665	764 (13.5)	1893	257 (13.6)	3772	253 (13.3)	
Class 2	5665	498 (8.8)	1893	181 (9.6)	3772	162 (8.5)	
Class 3	5665	1115 (19.7)	1893	359 (19.0)	3772	394 (20.6)	0.199
Class 4	5665	763 (13.5)	1893	234 (12.4)	3772	265 (13.9)	
Class 5	5665	1801 (31.8)	1893	602 (31.8)	3772	592 (31.0)	
Class 6 (the lowest)	5665	724 (12.8)	1893	260 (13.7)	3772	242 (12.7)	
Anthropometric data							
Weight (kg)	5768	31.2 (16.7)	1996	19.2 (13.2)	3772	37.3 (15.3)	<0.001
Height (cm)	5511	127.4 (30.4)	1739	101.5 (31.2)	3772	139.0 (21.0)	<0.001
BMI (z-score) [a]	4833	0.55 (1.70)	1061	0.47 (2.55)	3772	0.60 (1.40)	<0.001
Overweight/Obesity [a]	4744	1771 (37.3)	972	323 (33.2)	3772	749 (39.3)	0.003
Diet quality							
S-HEI (score)	5816	70.4 (9.5)	2044	71.5 (10.1)	3772	69.5 (9.1)	<0.001
High-quality diet	5816	1940 (33.4)	2044	683 (33.4)	3772	1257 (33.3)	
Medium-quality diet	5816	1968 (33.8)	2044	680 (33.3)	3772	1288 (34.1)	0.757
Low-quality diet	5816	1908 (32.8)	2044	681 (33.3)	3772	1227 (32.5)	
24-h movement guidelines							
PA guideline (%, yes)	6068	1600 (26.4)	2296	324 (14.1)	3772	1276 (33.8)	<0.001
ST guideline on weekdays (%, yes)	5832	4755 (81.5)	2060	1780 (86.4)	3772	2975 (78.9)	<0.001
ST guideline on weekends (%, yes)	5830	3514 (60.3)	2058	1496 (71.7)	3772	2018 (53.5)	<0.001

ST guideline globally (%, yes)	5827	3305 (56.7)	2055	1448 (70.5)	3772	2921 (77.4)	<0.001
Sleep duration guideline (%, yes)	6106	4522 (74.1)	2334	1601 (68.6)	3772	1857 (49.2)	<0.001
All the three guidelines (%, yes)	5806	660 (11.4)	2034	152 (7.5)	3772	508 (13.5)	<0.001
Socioemotional behavioral problems							
SDQ (score)	4604	7.4 (5.1)	832	7.3 (5.1)	3772	7.4 (5.1)	0.610
Normal	4604	4006 (87.0)	832	723 (86.9)	3772	3283 (87.0)	
Borderline	4604	314 (6.8)	832	55 (6.6)	3772	259 (6.9)	0.888
Abnormal	4604	284 (6.2)	832	54 (6.5)	3772	230 (6.1)	

BMI: Body mass index; HDI: Human Development Index; PA: Physical activity; S-HEI: Spanish Healthy Eating Index; SDQ: Strengths and Difficulties Questionnaire; SES: Socioeconomic status; ST: Screen time [a] According to the International Obesity Task Force criteria[23]
