# Supplementary material for: Breakfast and psychosocial behavioural problems in young population: The role of status, place, and habits
Source: Front Nutr. 2022 Aug 23;9:871238. doi: 10.3389/fnut.2022.871238 (PMC9445130; doi:10.3389/fnut.2022.871238)
Supplement: Supplementary file 2 [file Table_2.pdf]

Table S2. Criteria to establish the score for each item in the Spanish Health Eating Index.

Criteria to define the score for each item in the Spanish Health Eating Index (S-HEI)					
**Variables**	Criteria for a maximum score of 10	Criteria for a maximum score of 7.5	Criteria for a maximum score of 5	Criteria for a maximum score of 2.5	Criteria for a maximum score of 0
Daily					
Bread or grains	Daily	Three or more times per week, but not daily	Once or twice per week	Less than once per week	Never or almost never
Leafy greens, salads and vegetables	Daily	Three or more times per week, but not daily	Once or twice per week	Less than once per week	Never or almost never
Fresh fruit (excluding juices)	Daily	Three or more times per week, but not daily	Once or twice per week	Less than once per week	Never or almost never
Dairy products (milk, cheese, yoghurt)	Daily	Three or more times per week, but not daily	Once or twice per week	Less than once per week	Never or almost never
Weekly consumption					
Meat (chicken, beef, pork, lamb, etc.)	Once or twice per week	Three or more times per week, but not daily	Less than once per week	Daily	Never or almost never
Legumes	Once or twice per week	Three or more times per week, but not daily	Less than once per week	Daily	Never or almost never
Occasional consumption					
Cold meats and cuts	Never or almost never	Less than once per week	Once or twice per week	Three or more times per week, but not daily	Daily
Sweets (biscuits, pastries, jams, cereals with sugar, sweets, etc.)	Never or almost never	Less than once per week	Once or twice per week	Three or more times per week, but not daily	Daily
Soft drinks with sugar	Never or almost never	Less than once per week	Once or twice per week	Three or more times per week, but not daily	Daily
Variety	2 points if participant achieve each of the daily recommendations, 1 point if participant achieve each of the weekly recommendations.				

Each item ranged from 0 to 10 points, according to the criteria of the Spanish Health Eating Index (S-HEI)(1), where 10 points indicates that the the Spanish Society of Community Nutrition recommendations were met(2).

Supplementary references

1. Norte Navarro, A.; Ortiz Moncada, R. Spanish diet quality according to the healthy eating index. *Nutr. Hosp*. **2011**, *26*, 330–336, doi: 10.1590/S0212-16112011000200014.
2. Spanish Society of Community Nutrition (SSCN). Healthy dietary guidelines (2014). Available online: http://www.nutricioncomunitaria.org/es/otras-publicaciones (accessed on 13 December 2020).
